# Performance and clinical utility of two targeted multigene panels for GIST molecular characterization

**DOI:** 10.1038/s41598-025-30548-7

**Published:** 2025-12-17

**Authors:** Margherita Nannini, Annalisa Astolfi, Thais Maloberti, Maria Concetta Nigro, Livia Gozzellino, Alice Costa, Maria Giulia Pirini, Antonio De Leo, Marco Grillini, Annalisa Altimari, Massimo Del Gaudio, Bruno Vincenzi, Elena Fumagalli, Antonella Brunello, Giovanni Grignani, Sandra Aliberti, Angela Dalia Ricci, Fabio Gelsomino, Elisabetta Setola, Giovanni Tallini, Dario de Biase, Maria Abbondanza Pantaleo

**Affiliations:** 1 Department of Medical and Surgical Sciences (DIMEC), University of Bologna, Bologna, 40138 Italy; 2 Medical Oncology, IRCCS Azienda Ospedaliero-Universitaria di Bologna, Bologna, 40138 Italy; 3 IRCCS Azienda Ospedaliero-Universitaria di Bologna, Bologna, 40138 Italy; 4 Solid Tumor Molecular Pathology Laboratory, IRCCS Azienda Ospedaliero-Universitaria Di Bologna, Bologna, Italy; 5 Pathology Unit, IRCCS Azienda Ospedaliero-Universitaria di Bologna, Bologna, Italy; 6 Hepato-biliary and Transplant Unit, IRCCS Azienda Ospedaliero-Universitaria di Bologna, Bologna, Italy; 7 Department of Medical Oncology, Campus Biomedico University of Rome, Rome, Italy; 8 Medical Oncology Unit 2, Fondazione IRCCS, Istituto Nazionale dei Tumori, Milano, Italy; 9 Department of Oncology, Medical Oncology 1, Veneto Institute of Oncology IOV - IRCCS, Padua, Italy; 10 Candiolo Cancer Institute, FPO – IRCCS, St. Provinciale 142, Km 3.95, Candiolo (TO), 10060 Italy; 11 Medical Oncology Unit, National Institute of Gastroenterology, IRCCS “S. de Bellis” Research Hospital, Via Turi 27, Castellana Grotte, 70013 BA Italy; 12 Department of Oncology and Hematology, Division of Oncology, University Hospital of Modena, Modena, 41124 Italy; 13 Department of Medical Oncology, Istituto Europeo Oncologia, Milano, Italy; 14 Department of Pharmacy and Biotechnology (FaBit), University of Bologna, Bologna, Italy

**Keywords:** Gastrointestinal stromal tumor, GISTs, NGS, Targeted multigene panels, Molecular analysis, Cancer, Gastroenterology, Genetics, Oncology

## Abstract

Molecular analysis is mandatory in the diagnostic work-up of gastrointestinal stromal tumors (GISTs). Indeed, it is essential for clinical decisions, from patients’ selection for systemic treatment to identifying unrecognized syndromic conditions. Since GISTs are recognized as a heterogeneous family of different clinical entities, molecular analysis should also require a feasible, rapid, and reliable diagnostic workflow. Herein, we present our experience on the performance and clinical utility of two lab-developed multigene-NGS panels specifically built for GIST analysis. Among 163 analyzed GISTs, 72.4% carried *KIT* mutations while 11.0% were *PDGFRA*-mutant. Among putative *KIT*/*PDGFRA* WT cases that arrived at our attention from an external analysis, nine of 10 were found carrying either *KIT* or *PDGFRA* pathogenic mutations by our panel. On 26 *KIT/PDGFRA/BRAF* WT patients at the first level, the second level panel identified *NF1* or *SDHA* mutations in 16 cases, while 10 patients did not display any mutation, except for two of them found as carriers of SDHC epimutation. This optimized NGS diagnostic approach helps to characterize the molecular profiles of GIST and drastically reduces the number of truly non-KIT and non-PDGFRA-addicted GIST cases.

**Supplementary Information:**

The online version contains supplementary material available at 10.1038/s41598-025-30548-7.

## Introduction

The inclusion of molecular analysis of *KIT* and *PDGFRA* in the diagnostic work-up of gastrointestinal stromal tumors (GISTs) is considered standard clinical practice^1^. *KIT* and *PDGFRA* mutations have a proven pathogenetic role in GISTs and represent the main targets of tyrosine kinase inhibitors (TKIs) currently used in clinical practice^2,3^. The clinical relevance of molecular analysis has grown over time, showing profound implications that clearly affect the overall survival of GIST patients^4^. Indeed, GISTs are recognized worldwide as a heterogeneous family of different clinical entities with well-settled molecular features. Besides *KIT* and *PDGFRA* mutations, affecting about 90% of all GISTs, over the years, other rarer but clinically significant molecular alterations have been found^5–15^. Each molecular data has both predictive and prognostic values, essential for every clinical decision, from patients’ selection for systemic treatment to identifying unrecognized syndromic conditions^16–18^.

For this reason, in the past years, conventional Sanger sequencing and Real-Time PCR have been progressively replaced by multigene Next-Generation Sequencing (NGS) technologies, capable of identifying both frequent events at a low variant allele frequency and other genetic alterations, such as those in *SDHx*, *NF1*, and *BRAF* mutations^19^. Anyway, to date a laboratory test (commercial or not) that includes the most relevant genes in the management of GISTs (*KIT*,* PDGFRA*,* SDHx*,* NF1*,* BRAF*,* KRAS*,* FGFR1*) is not available in routine clinical practice, which leads either to missing important genetic information in GIST diagnostics, or to rely on multiple assays or techniques to complete the molecular assessment of the tumor. Moreover, since some GIST-relevant genes are challenging due to either large dimensions or the presence of many similar pseudogenes (*NF1*,* SDHx*), they are frequently excluded from commercial NGS panels for oncology. Actually, even if commercial oncology NGS panels generally include *KIT*, *PDGFRA*, and *BRAF* full or hotspot sequencing, no commercial assay targets SDH subunits along with *NF1* and *FGFR1*. Noteworthy, while *KIT* and *PDGFRA* and more recently *BRAF* mutations are notoriously targets of specific drugs currently used in clinical practice, the identification of *SDHx* and *NF1* mutations holds clinically relevant value for prognostic outcome prediction and therapeutic decision making, besides essential information on the need for genetic counselling and surveillance for the patient and close relatives.

To date, molecular analysis is mandatory for clinical practice, but should also require a feasible, rapid, and reliable targeted multi-gene test and a standardized diagnostic workflow^20^.

This study aimed to develop and validate two laboratory-designed multigene NGS panels specifically optimized for GIST molecular assessment, addressing current limitations of commercial assays by improving coverage of challenging genomic regions and enabling comprehensive, clinically relevant mutation profiling in a single workflow.

## Materials and methods

### Patients

We included patients affected by GIST managed in different centers, expert or not in GIST, and arrived at our attention for molecular testing using lab-developed multi-gene panels. All pathological, clinical, and follow-up data were anonymously collected from in- and out-patient medical records in an electronic database. Confirmed written consent for molecular testing was obtained from all patients.

The study was performed in accordance with the Declaration of Helsinki protocols. The study was reviewed and approved by the local Institutional Ethical Committee of Azienda Ospedaliero-Universitaria Policlinico S. Orsola-Malpighi, Bologna, Italy (approval number 113/2008/U/Tess), and informed consent was provided by all living patients.

### Molecular analysis

All analyzed tumor samples were formalin-fixed and paraffin-embedded (FFPE). DNA was extracted from two to three 10-um-thick sections, according to the selection performed by a pathologist on the last Hematoxylin and Eosin (H/E) slide. The DNA was then quantified using a Qubit fluorometer (Thermo Fisher Scientific, Waltham, MA, USA).

Sequencing was performed using two multi-gene NGS panels developed in the Molecular Pathology Laboratory of Solid Tumors - IRCCS Policlinico di S.Orsola, allowing the analysis of the following genomic regions.

*Panel 1* (“first-level” panel, 229 amplicons, 15.04 kb, human reference sequence hg19/GRCh37)^11^: ***BRAF*** (exons 11, 15), *CTNNB1* (exon 3), *EGFR* (exons 12, 18, 19, 20, 21), *EIF1AX* (exons 1, 2), *GNA11* (exons 4, 5), *GNAQ* (exons 4, 5), *GNAS* (exons 8, 9), *H3F3A* (exon 1), ***HRAS*** (exons 2, 3), *IDH1* (exon 4), *IDH2* (exon 4), ***KIT*** (exons 8, 9, 11, 13, 17), ***KRAS*** (exons 2, 3, 4), *MED12* (exons 1, 2), *MET* (exons 2, 14), *MYC* (exons 1–3), ***NRAS*** (exons 2, 3, 4), ***PDGFRA*** (exons 12, 14, 18), *PIK3CA* (exons 10, 21), *PTEN* (exon 5), *RET* (exons 5, 8, 10, 11, 13, 15, 16), *RNF43* (exons 2, 8), *SMAD4* (exons 6, 9, 10, 11, 12), *TERT* (promoter region, g.1295141–g.1295471), and *TP53* (exons 4, 5, 6, 7, 8, 9).

*Panel 2* (“second-level” panel, 276 amplicons, 23.02 kb, human reference sequence hg19/GRCh37): *SDHA* (entire coding sequence - CDS), *SDHB* (CDS), *SDHC* (CDS), *SDHD* (CDS), *NF1* (CDS), *FGFR1* (CDS). According to previous validation^21^, only mutations present in at least 5% of the total number of reads analyzed and observed in both strands were considered for mutational calls. The Varsome (https://varsome.com/) and Franklin by Genoox (https://franklin.genoox.com/clinical-db/home*)* tools were used to evaluate the ACMG classification of each reported variant (last accessed 20th August 2024). Only Pathogenic/Likely Pathogenic and VUS (Variant of Uncertain Significance) variants were reported.

### Statistical analysis

Comparison between main clinic-pathological features (gender, age, primary tumor site) and molecular subgroups was performed with either Unpaired t-test or Pearson’s chi-square statistics, by using IBM SPSS Statistics for Windows Version 19.0 (IBM Corporation, Armonk, NY, USA).

## Results

A total of 163 GIST patients were included, of which 153 (93.9%) underwent molecular analysis upfront in our laboratory, while 10 (6.1%) were sent from other laboratories as initially suspected *KIT/PDGFRA* WT cases. Overall, 136 patients (83.4%) underwent lab-developed “first-level” panel only, whereas the remaining 27 (16.6%) underwent both “first-” and “second-level” panels. Tumor and patients’ characteristics are reported in Table 1.

**Table 1:** Clinic-pathological characteristics.

Total 163		
	Median (range) yy	*N* (%)
**Age**	61 (23–87)	
**Gender** F M		91 (55.8) 72 (44.2)
**Primary tumor site** stomach duodenum digiunum ileum rectum extra-GIST colon NA		84 (51.5) 14 (8.6) 13 (7.8) 43 (26.4) 3 (1.8) 3 (1.8) 1 (0.6) 2 (1.2)
**Disease status_diagnosis** Localized Advanced NE		122 (74.9) 37 (22.7) 4 (2.4)
**Risk stratification** Null Very low Low Intermedium High NE NA		6 (3.7) 26 (15.9) 33 (20.2) 20 (12.3) 30 (18.4) 11 (6.7) 37 (22.7)

NE = not evaluable; NA = not applicable (advanced disease up-front);.

Among 136 patients studied with the “first-” panel only, *KIT* mutations were found in 118 cases (72.4%), and *PDGFRA* mutations were detected in 18 cases (11.0%).

**Fig. 1:**
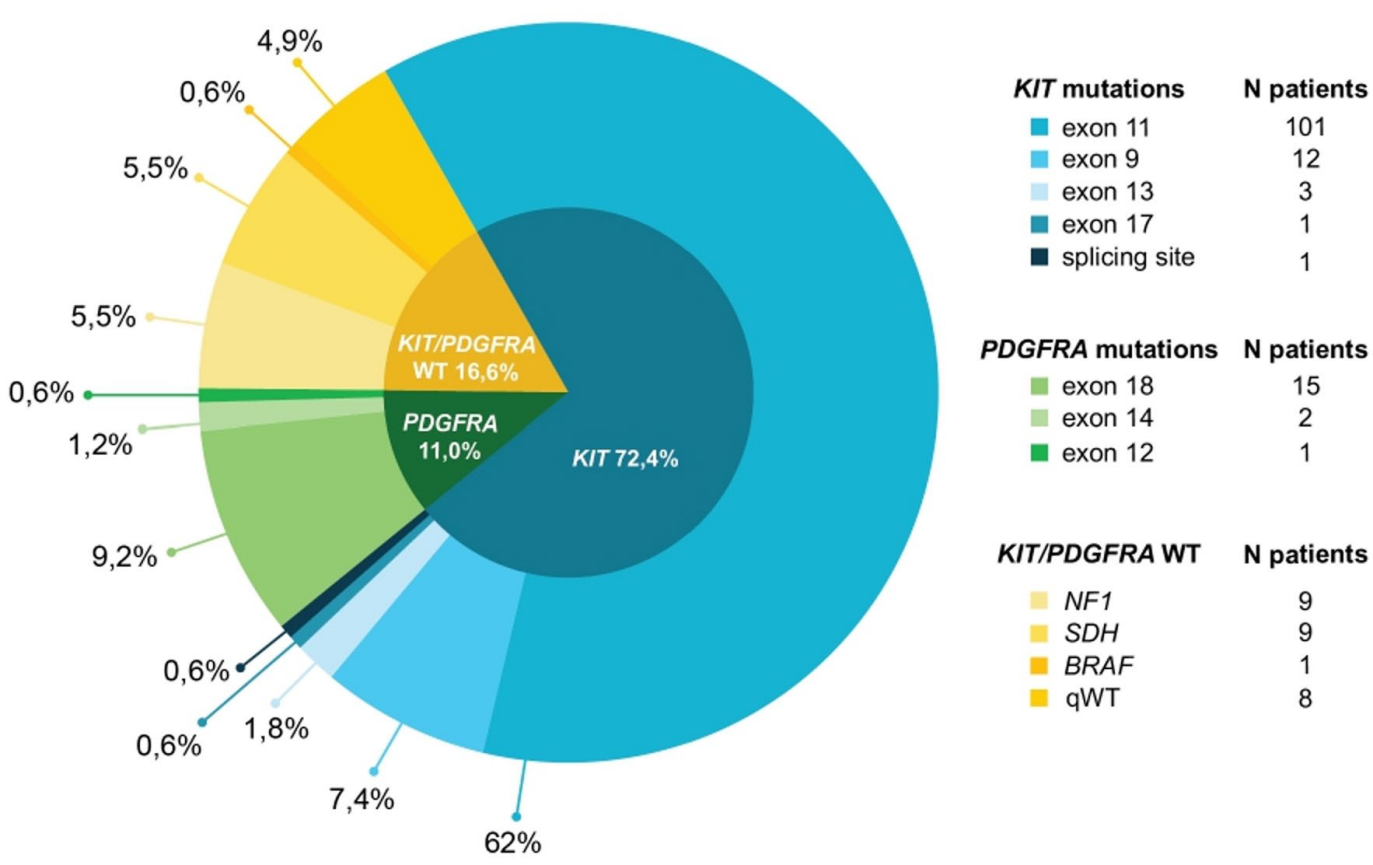
Type and frequency of mutations found by first-level and second-level panel.

In particular, for *KIT* mutations: 85.6% in exon 11, 10.2% in exon 9, 3.9% in exon 13, 1.7% in exon 17, and 0.6% in the splicing site (Fig. 1).

**Fig. 2:**
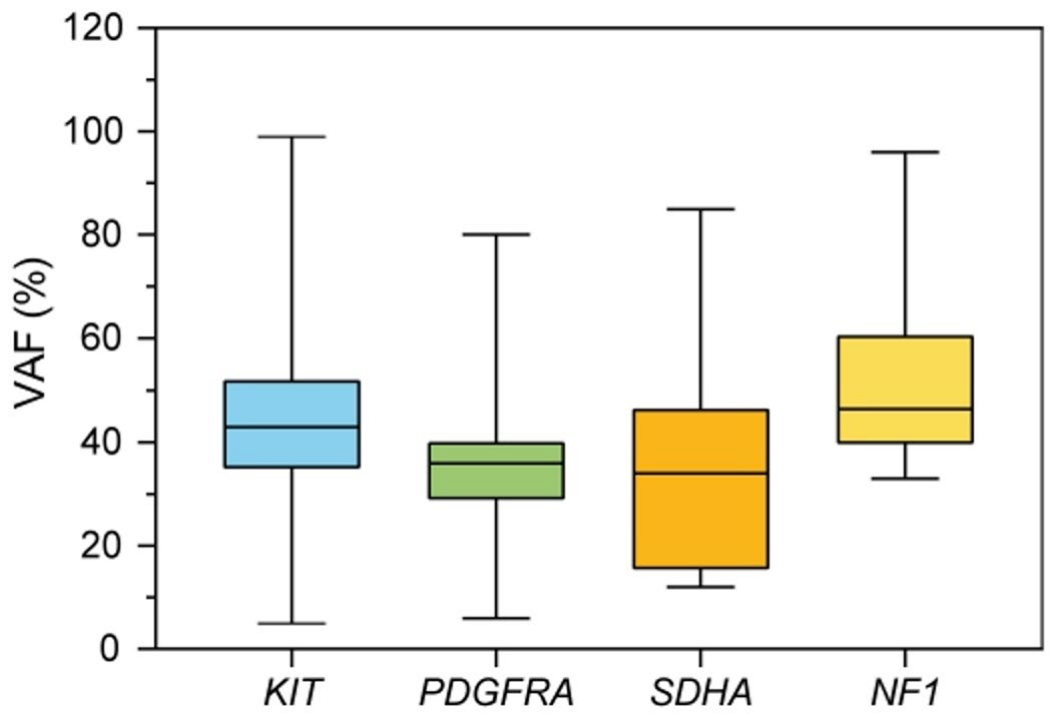
Box plot rapresentation of detected VAF.

A summary of the distribution and most frequent types of *KIT* variants is listed in Supplementary Table 1. In three cases, biallelic or triallelic *KIT* mutations were detected. The Variant Allele Frequency (VAF) for all detected mutations ranged from 5% to 99% (mean 45.1%) (Fig. 2).

All the detected mutations were pathogenic/likely pathogenic variants, except for two (exon 11: c.1730_1774 + 3dup; exon 9: p.Ser451Cys), classified as “variant of uncertain significance - VUS”. However, they were considered pathogenetic according to the type of mutation and patients’ clinical features.

For *PDGFRA* mutations: 83.3% in exon 18, 11.1% in exon 14, and 5.6% in exon 12 (Fig. 1). All mutations were pathogenic/likely pathogenic variants, with a VAF ranging from 6% to 80% (mean 34.7%) (Fig. 2). The distribution of *PDGFRA* variant types is listed in Supplementary Table 1.

Notably, 90% (9 out of 10) cases referred to as putative *KIT/PDGFRA* WT from an external analysis resulted in *KIT* or *PDGFRA* mutations already at the first-level panel performed for validation in our laboratory (Table 2).

**Table 2:** List of cases referred as putative *KIT/PDGFRA* WT from an external analysis. All cases exhibited pathogenic variants in *KIT/PDGFRA/BRAF* with our first-level panel.

Code	Gender	Age	Tumor_site	Disease status/ class risk	Gene	Exon	*p*.	c.	VAF (%)	First Technique
PAN_6	F	51	Ileum	Localized/High	***BRAF***	15	p.Val600Glu	c.1799T > A	44	Sanger sequencing KIT/PDGFRA
PAN_12	F	63	Stomach	Localized/High	***KIT***	11	p.Pro577_Gly592dup	c.1728_1775dup	37	Commercial NGS kit
PAN_29	M	55	Stomach	Localized/High	***KIT***	11	p.Val555_Pro573del	c.1663_1719del	30	Commercial NGS kit
PAN_36	M	35	Rectum	Localized/High	***KIT***	11	p.Trp557_Lys558del	c.1669_1674del	40	Real-Time PCR
PAN_69	F	80	Ileum	Advanced	***KIT***	Splice Site	/	c.1648-4_1670del	75	Real-Time PCR
PAN_101	M	66	Stomach	Localized/Intermedium	***KIT***	11	p.Asp572_Ser590dup	c.1714_1770dup	20	Commercial NGS kit
PAN_102	M	60	Digiunum	Localized/High	***KIT***	11	p.Gln575_Pro577delinsThr	c.1723_1729delinsA	42	Real-Time PCR
PAN_103	F	62	Stomach	Localized/Low	***PDGFRA***	18	p.Asp842_Ile843delinsVal	c.2525_2527del	80	Real-Time PCR
PAN_105	F	34	Stomach	Localized/Intermedium	***KIT***	11	p.Pro573_Asn587dup	c.1717_1761dup	5	Commercial NGS kit
PAN_106	M	54	Stomach	Localized/High	***KIT***	11	p.Pro577_Ser590dup	c.1729_1770dup	28	Commercial NGS kit

Many of these previously undetected mutant cases were carriers of complex genetic events, including large rearrangements (mainly duplications) or deletions spanning the intron 10/exon 11 boundary (6 cases), while one harbored a low-allele-fraction mutation. For most of them, this finding led to a significant therapeutic change, especially in considering imatinib adjuvant therapy in high-risk cases, with a potential impact on overall prognosis. In the other case (1 out 10), a *BRAF* p.Val600Glu mutation was detected using the first-level panel. Conversely, no alterations were detected in the *RAS* (*KRAS*, *HRAS*, *NRAS*) genes. Overall, the first-level panel successfully identified mutations in 12 cases with low-allele-fraction variants (VAF < 20%; mean: 13%), which would likely have been missed by Sanger sequencing due to its lower detection limit. Moreover, in 13 cases, it also identified very large alterations such as duplications, deletions, or insertions larger than 30 nucleotides, confirming the sensitivity not only of the test but also of the mapping algorithm.

Among 26 patients who required both “first-” and “second-level” panels because they resulted *KIT/PDGFRA/BRAF* WT from the first-level panel, a further second-level panel analysis allowed us to identify other gene alterations in 16 patients. In particular, *NF1* mutations were found in 9 cases (5.5% of the entire cohort) (Fig. 1), with a VAF ranging from 10% to 96% (mean = 54.7%) (Fig. 2). *SDHA* mutations were found in 7 cases (4.3% of the entire cohort), with a VAF ranging from 12% to 85% (mean 38.9%) (Fig. 2), thus confirming the high prevalence of subunit A mutation in SDH*-deficient* GIST. In two cases, harboring pathogenetic *SDHA* mutations, a *TP53* co-mutation was detected.

In 10 patients (6.1% of the entire cohort), no gene alterations, either by first or second level panel analysis, were found. At a further deeper analysis of two cases with clinical features belonging to *SDH*x-*deficient* GIST, an expected *SDHC* promoter methylation was discovered (Fig. 1). Therefore, in only 8 patients (4.9% of the entire cohort), with morphological and immunohistochemical features consistent with the diagnosis of GIST, no gene alterations have been found (Fig. 1).

In order to evaluate the ability of the second-level panel to identify *SDHx* mutations, 21 known SDH-*deficient* cases already characterized by Sanger sequencing were analyzed for internal validation. By second-level analysis, *SDHx* mutations were found in all cases, thus confirming the high diagnostic sensitivity of the panel, especially for *SDHA* mutations that are more challenging to identify due to the presence of highly similar pseudogenes. The analysis identified multiple types of *SDH*x mutations, with a vast majority of missense and nonsense variants (22 and 10, respectively), but also frameshift ins/del and splice-site mutations (6 cases).

**Fig. 3:**
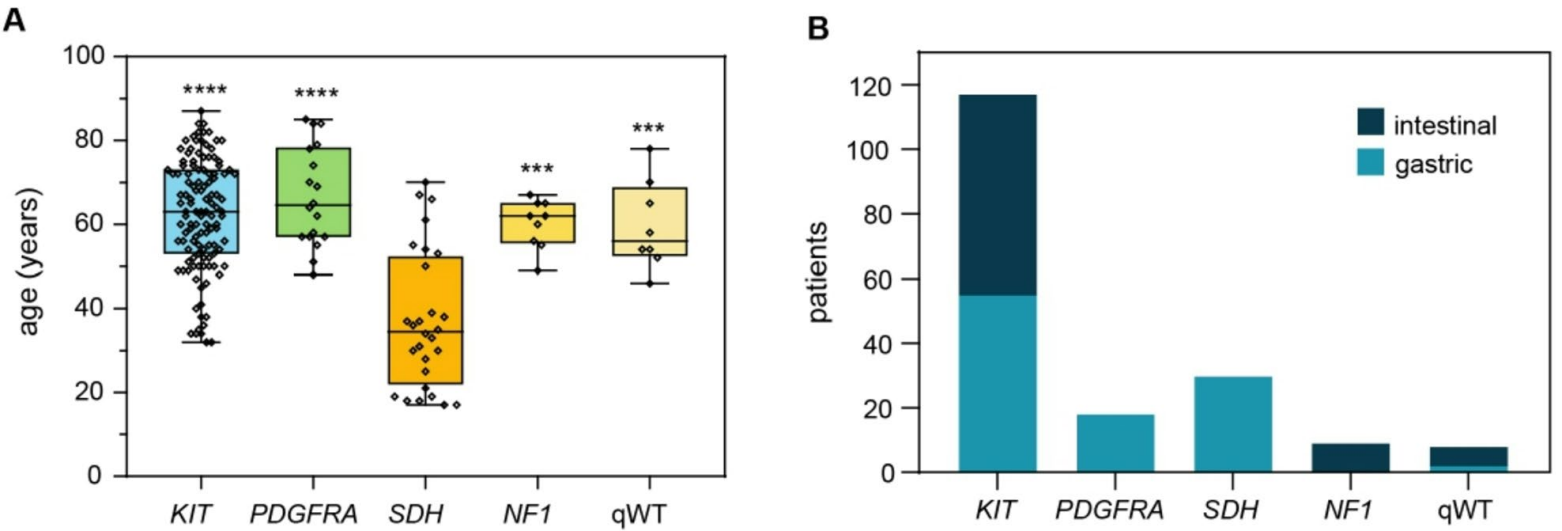
**A**: Age distribution according to molecular status; **B**: primary site distribution according to molecular status.

Looking at the entire cases included in the study, a correlation between clinical-pathological features and tumor genotype has been confirmed. In particular, a statistical difference in median age at diagnosis (*p* = 0.0001) and primary tumor site (*p* = 0.0001) was found, with SDH-*deficient* GISTs mostly affecting young-adult patients (mean age at diagnosis: 37 ± 3 years) with exclusive gastric localization (30/30) (Fig. 3A-B). Conversely, while *KIT* mutant cases showed an equal distribution in primary tumor site, *NF1*-mutant cases had an exclusive small intestine localization (9/9), and *PDGFRA*-mutant cases had gastric localization only (18/18) (Fig. 3B).

## Discussion

Mutational analysis incorporation in the diagnostic work-up of all GISTs should be considered standard practice, due to its undoubted predictive value for disease classification, prediction of sensitivity to molecular-targeted therapies, as well as a prognostic relevance^1^. Thus, centralization of mutational analysis in a laboratory enrolled in an external quality assurance program and with high expertise in the disease may be relevant, especially for those cases without typical molecular alterations^1^. The lack of *KIT* and *PDGFRA* mutations, which once was enough to classify GISTs as WT, nowadays deserves to be interpreted in the specific patient clinical context and, if needed, questioned in case of genotype-phenotype clinical consistency^19^. Secondly, it then requires further analysis for the identification of other alterations, which are currently actionable or allow to identify those GISTs underlying unrecognized syndromes^17,18^.

In the present study, we reported the performance of two lab-developed multigene panels specifically built for GIST molecular analysis with a two-step approach to establish a standardized analytical workflow useful in clinical practice.

As expected, in 83.4% of cases first-level panel allowed the identification of pathogenetic alterations. Among patients undergoing second-level analysis, *NF1* and *SDHx* mutations were found in more than half of the cases. Thus, excluding those two cases with *SDHC* methylation, in 93.9% of GISTs, at least one gene alteration by both panels has been found.

Noteworthy, all patients referred as *KIT/PDGFRA* WT from external analyses, which underwent the first-level panel for validation, were found to be mutated, including one case with a *BRAF* p.V600E mutation (Table 2). In this latter case (PAN_6, Table 2), the external institution had only performed *KIT* and *PDGFRA* analysis using Sanger sequencing, and for this reason, it had not been possible to identify the *BRAF* mutation. In 4 cases (PAN_36, PAN_69, PAN_102, PAN_103 - Table 2), the initial analysis was performed using Real-Time PCR, and it is known that the use of mutation-specific methods could lead to false negative results if the variants present in the sample are not included in the set of mutations identifiable by the kit in use. The other cases initially classified as WT (PAN_12, PAN_29, PAN_101, PAN_105, PAN_106 - Table 2) were analyzed in external institutions using NGS kits. However, these undetected mutant GIST cases included both very large rearrangements (duplications or deletions of more than 42 nucleotides) or intron 10/exon 11 spanning deletions or low-allele-fraction mutations. To ensure the accuracy of the results obtained using our lab-developed panel and to rule out possible false positives, the analysis was performed twice. Furthermore, the positivity of these cases was also confirmed by the clinical course of the patients. These results confirm previous reports on the most frequently missed *KIT* mutations in GISTs and support the need to refer patients with putative *KIT/PDGFRA* WT GIST to highly specialized molecular diagnostic centers that implement appropriate NGS panels and bioinformatic pipelines to detect even complex variants^19^. Indeed, the frequency of complex or large alterations and of low-allele-fraction mutations is high (18 cases carrying at least one of the two; 13.2% of all *KIT/PDGFRA*-mutant patients), thus further highlighting the need for centralization of GIST molecular diagnosis at least for WT cases, since these alterations would have probably been missed by routine molecular analyses.

The performance of the second-level panel in the detection of SDHx-mutations was validated on additional 21 SDHx-*deficient* GISTs, confirming the high diagnostic sensitivity of the panel (100% of mutations correctly identified), especially for the detection of *SDHA* mutations that are more challenging to identify due to the presence of highly similar pseudogenes. Actually, few *SDHA* mutations were classified as VUS, probably due to the still low frequency of these alterations and to the absence of hotspot mutation domains, which is the typical mutational profile expected for tumor suppressor genes. In these cases, coupling NGS panel sequencing with SDHB immunohistochemistry should help to identify SDH-*deficient* GISTs^4,22–24^. Moreover, clinical features should always guide the need for further molecular testing: in fact, we showed that two patients classified as WT from first and second-level panel analyses but showing typical features of SDH-*deficient* GISTs were confirmed as carriers of *SDHC* epimutations. Therefore, in agreement with data shown in the literature, the concordance between genotype and phenotype has been confirmed, highlighting once again the importance of clinical features to interpret unusual or unexpected molecular alterations or when typical alterations are not found^6,7,10–12^.

SDH-*deficiency* should always be investigated in younger patients, especially females, with gastric primary GIST and without conventional gene mutations. This is also valid when *SDH*x mutations with uncertain significance or when no *SDH*x mutations are found, recommending the implementation of immunohistochemistry (IHC) for SDH complex subunit B (SDHB) and subunit A (SDHA) in the diagnostic workflow^25^. Conversely, small intestine primary GISTs, generally multifocal, without *KIT* and *PDGFRA* mutations, should raise suspicion of an NF1-related GIST, even when the pathognomonic NF1 clinical features are lacking^17^. In these *KIT/PDGFRA* WT cases without a clear diagnosis of NF1 syndrome but just suspected for multifocality and site localization of GIST, the multigene first-level panel could be more useful. Conversely, for *KIT/PDGFRA* WT cases with clinical features of a genetic disease, the multigene second-level panel could help to characterize the NF1-related GIST and to make a diagnosis of neurofibromatosis genetic disease. The e of NF1-related GIST in our series was 9 out of 26 *KIT/PDGFRA* wild-type cases (5.5% of the entire cohort). The detection rate of *NF1*-related GIST in our series was higher than expected from previous reports^6^, even if a very recent NGS-based approach reached a similar high frequency (16/35)^26^. These discrepancies could reflect either the expected fluctuations due to the small numbers of a rare disease, the possible higher efficacy of our panel in detecting variants in a very challenging gene (*NF1* is very large and with known similar pseudogenes), or lastly, the possibility that more difficult GIST molecular diagnoses were prevalently directed to our specialized center. Importantly, given the established associations of *NF1* and *SDHx* variants with hereditary tumor syndromes, the identification of either SDH-*deficient* or NF1-related GISTs should always prompt genetic counseling to guide appropriate genetic testing, interpret variants of uncertain significance, and provide information for patient and family risk assessment and management.

Notably, in our series, only 4.9% of cases were truly negative (*KIT*/*PDGFRA*/*BRAF*/*NF1*/*SDH*x). In these cases, there is a need to widen and deepen the molecular analyses, since it is known that quadruple WT GIST may have very heterogeneous mutational profiles, with agnostic relevance in case of NTRK-fusion positive GISTs^4,27,28^. We therefore suggest that this very rare molecular condition should be profiled by a comprehensive sequencing assay, to uncover unexpected or rare conditions, that are usually not included in routine NGS panel analyses.

**Fig. 4:**
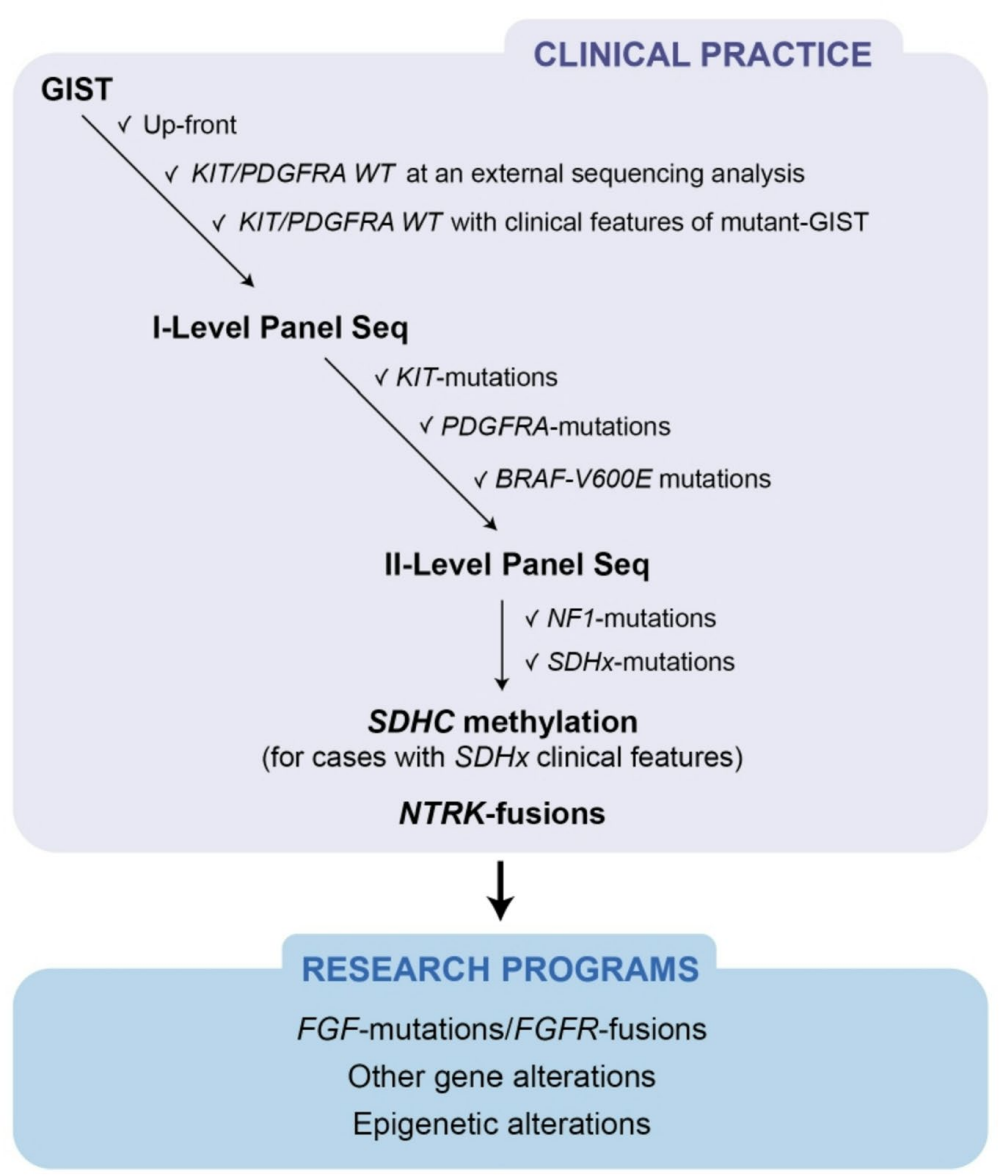
Sequential workflow of routine molecular assessment in GISTs Therefore. based on our clinical and molecular experience, the routine molecular assessment in GIST could follow a sequential workflow, that firstly explores KIT/PDGFRA mutations which are expected in more than 80% of GISTs, thus speeding up the molecular diagnostic assessment of the disease and lowering the economic burden, being able to analyze in the same run also other solid tumors that are covered by the same NGS panel., (Fig. 4).

Conversely, the second-level panel is restricted only to the lower number of cases that remain negative after the first-level analysis. In our hands, this approach is able to save time and money, even if a truly dedicated GIST panel can indeed be designed that merges the two gene lists.

In conclusion, in clinical practice, an additional double-check of the molecular analysis in high-volume reference centers is still crucial, even just for *KIT* and *PDGFRA* genes. This approach lowers the percentage of *KIT/PDGFRA* WT GIST from the previously known 10–15% to less than 5% and this result is extremely important in any context. Furthermore, in specialized centers, the expertise of the multidisciplinary team can benefit from the application of the suggested diagnostic workflow which combines a high-performance laboratory test with the experienced clinical assessment of patients, thus being able to classify GIST according to all known molecular subgroups (*KIT*/*PDGFRA*/*BRAF*/*NF1*/*SDH*x mutant). In practice, this optimized sequential approach helps to characterize the molecular profiles of GISTs and drastically reduces the number of truly oncogene-negative GIST cases which undoubtedly deserve further molecular screening within research programs.

## Supplementary Information

Below is the link to the electronic supplementary material.

Supplementary Material 1

## Data Availability

The datasets used and/or analysed during the current study available from the corresponding author on reasonable request.
