# Supplementary material for: Performance and clinical utility of two targeted multigene panels for GIST molecular characterization
Source: Sci Rep. 2025 Dec 17;16:855. doi: 10.1038/s41598-025-30548-7 (PMC12780020; doi:10.1038/s41598-025-30548-7)
Supplement: Supplementary file 1 — Supplementary Material 1 [file 41598_2025_30548_MOESM1_ESM.pdf]

Supplementary Table 1: Spectrum of *KIT* and *PDGFRA* gene alterations

	**N (%)**
KIT	Tot: **118**
Duplications	21 (17.8)
SNV	43 (36.4)
Ins/Del	54 (45.8)
p.Val559Asp	11 (9.3)
p.Ala502_Tyr503dup	10 (8.5)
p.Trp557Arg	9 (7.6)
p.Trp557_Lys558del	8 (6.8)
p.Trp557_Val560delinsPhe	5 (4.2)
other Duplications	11 (9.3)
other SNV	23 (19.5)
other Ins/Del	41 (34.7)
PDGFRA	Tot: **18**
SNV	15 (83.3)
Ins/Del	3 (16.6)
p.Asp842Val	10 (55.5)
other SNV	5 (27.7)

SNV: Single Nucleotide Variant; Ins/Del: Insertion/Deletion.
